# Clinical application of targeted next-generation sequencing in detecting organ/space surgical site infection in patients following biliary-pancreatic surgery

**DOI:** 10.3389/fmicb.2026.1866221

**Published:** 2026-06-26

**Authors:** Yifei Yang, Rong Jiang, Yichen Duan, Zhiang Wang, Yucheng Xu, Neng Tang, Yudong Qiu, Liang Mao, Xu Fu

**Affiliations:** 1 Department of Pancreatic and Metabolic Surgery, Nanjing Drum Tower Hospital, Affiliated Hospital of Medical School, Nanjing University, Nanjing, China; 2 Jiangsu Health Vocational College, Nanjing, China; 3 Department of Critical Care Medicine, Jinling Hospital, Affiliated Hospital of Medical School, Nanjing University, Nanjing, China

**Keywords:** biliary-pancreatic surgery, conventional microbiological tests, pathogen detection, surgical site infection, targeted next-generation sequencing

## Abstract

**Background:**

Organ/space surgical site infection (SSI) is a common and severe complication following biliary-pancreatic surgery. Rapid and accurate diagnosis is crucial for managing organ/space SSI. The clinical application of targeted next-generation sequencing (tNGS) in biliary-pancreatic surgery remains limited.

**Methods:**

This retrospective study enrolled 73 consecutive patients who underwent biliary-pancreatic surgery between January 2025 and June 2025, among whom 27 patients were clinically diagnosed with organ/space SSI. Abdominal drainage specimens were collected and analyzed using tNGS and conventional microbiological tests (CMTs). The diagnostic performance, pathogen spectrum, and turnaround time of the two methods were evaluated.

**Results:**

The overall pathogen detection rate was higher for tNGS than for CMTs (52.1% vs. 39.7%). tNGS showed higher sensitivity (92.6% vs. 66.7%) and negative predictive value (94.3% vs. 79.5%) than CMTs in organ/space SSI, with a shorter turnaround time (28.1 h vs. 109.1 h). tNGS revealed a broader pathogenic spectrum than CMTs (23 vs. 12 species). Moreover, advanced age (OR = 1.052, 95%CI = 1.005–1.100 *p* = 0.029) and preoperative biliary drainage (PBD) (OR = 9.411, 95%CI = 1.060–83.524, *p* = 0.044) were associated with tNGS positivity in exploratory multivariate analysis.

**Conclusion:**

tNGS may provide rapid adjunctive microbiological information for early etiological assessment of organ/space SSI after biliary-pancreatic surgery, but it should complement rather than replace CMTs and clinical assessment.

## Introduction

Organ/space surgical site infection (SSI) is a predominant and severe postoperative complication following biliary-pancreatic surgery, with reported incidence rates ranging from 13% to over 46% (Ellis et al., 2023; Feier et al., 2025; Kitagawa et al., 2025; Yang et al., 2025). The inherent complexity of biliary-pancreatic surgery, often involving cholangiojejunostomy or pancreaticojejunostomy, exposes patients to endogenous intestinal flora, which may contribute to the development of organ/space SSI (Rogers et al., 2017; Merali et al., 2024). The microbial profile of organ/space SSI following biliary-pancreatic surgery is typically dominated by enteric and biliary microorganisms, such as *Klebsiella pneumoniae*, *Escherichia coli*, and Enterococcus species (Belmouhand et al., 2018; Yang et al., 2021, 2024a,b). These organisms may originate from endogenous gastrointestinal flora, colonized bile, or even retrograde contamination through indwelling drainage systems. Our previous study identified *Klebsiella pneumoniae* in postoperative drainage fluid after pancreaticoduodenectomy as a clinically relevant pathogen associated with adverse postoperative outcomes (Yang et al., 2021). The occurrence of infection may trigger life-threatening secondary complications, prolong hospital stays, and even worsen oncological prognosis (Yang et al., 2024a,b). Therefore, early identification of clinically relevant pathogens may support etiological assessment and antimicrobial decision-making in patients with suspected organ/space SSI.

In clinical practice, organ/space SSI is diagnosed through a comprehensive evaluation of clinical signs, inflammatory markers, radiological findings, intraoperative or interventional evidence, and microbiological results when available (Bilgiç et al., 2020). Conventional microbiological tests (CMTs) of abdominal drainage fluid are commonly used as an adjunctive approach to obtain microbiological evidence in patients with suspected organ/space SSI. However, postoperative abdominal drainage fluid does not represent a completely sterile sampling environment, and microbiological findings from drainage fluid may be affected by drain colonization or external contamination. These results need to be interpreted in conjunction with the overall clinical context rather than being used as the sole basis for diagnosing organ/space SSI. In addition, CMTs are limited by relatively low sensitivity, and difficulty in recovering fastidious, anaerobic, or polymicrobial organisms (Flurin et al., 2021; Zhang et al., 2026). Moreover, CMTs typically require several days to yield pathogen identification and antimicrobial susceptibility results, thereby may delay targeted therapy and prolong empirical broad-spectrum antimicrobial therapy.

Recent developments in molecular diagnostics, particularly nucleic acid sequencing-based methods, offer a promising alternative to culture-based testing. Although metagenomic next-generation sequencing (mNGS) has been explored for identifying pathogens in respiratory and neurological infections (Gu et al., 2021; Rong et al., 2026), its application in postoperative abdominal drainage-fluid specimens from patients who underwent biliary-pancreatic surgery is still constrained by significant host DNA interference, prohibitive costs, and challenges in distinguishing true pathogens from transient background colonization. Targeted next-generation sequencing (tNGS), which amplifies specific genomic regions of pre-selected pathogenic panels rather than the entire microbiome, may provide a more focused and cost-effective approach for complex clinical specimens with low microbial loads (Rong et al., 2026). Although tNGS has shown diagnostic utility in respiratory tract infections and bloodstream infections (Li et al., 2022; Xu et al., 2024), its role in evaluating postoperative organ/space SSI following biliary-pancreatic surgery remains unexplored.

In the current study, we conducted a retrospective cohort study to evaluate the clinical application of tNGS compared to CMTs in detecting organ/space SSIs, describe the detected microbial spectrum, and explore factors associated with tNGS positivity in patients who underwent biliary-pancreatic surgery.

## Materials and methods

### Patients

This single-center, retrospective study was conducted at the Department of Pancreatic and Metabolic Surgery, Nanjing Drum Tower Hospital, Affiliated Hospital of Medical School, Nanjing University, including patients who underwent biliary-pancreatic surgery between January 2025 and June 2025. In our institution, postoperative abdominal drainage fluid was routinely collected for microbiological surveillance on postoperative day (POD) 1, 3, 5, and 7 in patients with abdominal drains. The inclusion criteria were as follows: (a) patients who underwent biliary or pancreatic surgery; (b) both tNGS and CMTs of postoperative drainage fluid were available, and (c) complete clinical data. The exclusion criteria were as follows: (a) patients with severe immunosuppressive systemic disease; (b) drainage fluid samples that did not meet the quality standards required for tNGS and/or CMTs; (c) clinical data were incomplete and (d) age < 18 years. The study was approved by the Health Research Ethics Board of Drum Tower Hospital of Nanjing University Medical School (2024-795-01) and conducted in accordance with the Declaration of Helsinki.

### Specimen collection and detection methods

Postoperative abdominal drainage-fluid specimens were collected according to the institutional postoperative surveillance protocol. Drainage fluid was routinely collected on POD 1, 3, 5, and 7 for CMTs. For tNGS, the POD 1 drainage fluid specimen was used to evaluate early pathogen detection. For patient-level analysis, tNGS positivity was defined according to the POD 1 tNGS result, whereas CMTs positivity was defined as at least one positive CMTs result from drainage-fluid specimens collected during POD 1, 3, 5, and 7. Samples were collected according to standardized protocols to minimize external contamination. Briefly, the drainage tube was disinfected with 75% alcohol and allowed to dry before sample collection. Approximately 15 mL of fresh, midstream abdominal drainage fluid was carefully obtained and transferred into sterile containers. In patients with multiple drainage tubes, fluid from each tube was collected separately and then combined for subsequent testing. All samples were promptly sent for culture and sequencing analysis.

For CMTs, clinical specimens were inoculated into both aerobic and anaerobic blood culture bottles, following previously reported protocols (Yang et al., 2025), and were processed using the BACT/ALERT VIRTUO automated blood culture system. Upon detection of a positive growth signal, the sample was extracted from the culture bottle for Gram staining or Acid-fast staining, followed by microscopic examination. Positive samples were then subcultured onto Columbia blood agar and MacConkey agar plates and incubated at 37 °C under 5% CO₂ atmosphere. Bacterial colonies grown on the media were further identified using matrix-assisted laser desorption/ionization time-of-flight mass spectrometry (MALDI-TOF MS) with the VITEK MS system (bioMérieux, France).

For tNGS, total nucleic acids of drainage fluid samples were extracted utilizing the GenK^©^ Magnetic Bead-based DNA/RNA Extraction Kit, followed by the reverse transcription of RNA to enable the concurrent detection of both DNA and RNA pathogens. Pathogen detection was performed using the Jingchayuan PISA targeted high-throughput sequencing assay (Genskey Medical). This assay is designed for multiple clinical specimen types, including pleural/ascitic fluid and tissue specimens, and targets a predefined panel of clinically relevant pathogens. The targeted pathogen panel covered microorganisms commonly encountered in biliary-pancreatic organ/space SSI (Yang et al., 2021, 2024a,b, 2025), including Enterobacteriaceae, particularly *Klebsiella pneumoniae* and *E. coli*, Enterococcus species, Staphylococcus species, anaerobic bacteria, and Candida species. Viral targets were reported when detected but were not automatically considered causative pathogens of organ/space SSI. The prepared libraries were sequenced using the MGISEQ-2000 platform to generate 50-bp single-end reads. Raw sequencing data were processed through rigorous quality control using fastp (version 0.23.2), ensuring a minimum of 1 Mb of clean reads with a quality score (Q30) > 85%. To minimize host DNA interference, the retained clean reads were mapped against the human reference genome (T2T-CHM13v2.0) using bowtie2 (version 2.3.5.1). The remaining non-host sequences were mapped to a comprehensive pathogen reference database via bwa-mem for taxonomic assignment, culminating in the generation of a comprehensive pathogen detection report.

### Clinical data collection and definition of complications

We retrospectively retrieved baseline demographic characteristics, intraoperative parameters, laboratory test on POD 1, and postoperative complications from the electronic medical record system of our institution. The diagnosis of organ/space SSI was established in accordance with the criteria issued by the Centers for Disease Control and Prevention (CDC) (Bilgiç et al., 2020). Briefly, organ/space SSI was defined as an infection occurring within the appropriate postoperative surveillance period, involving organ/space tissues deeper than the fascia or muscle layer, and meeting at least one of the following criteria within 30 days postoperatively: (1) purulent drainage from a drain placed into the organ/space; (2) organisms identified from fluid or tissue in the organ/space by culture- or non-culture-based microbiological testing performed for clinical diagnosis or treatment; (3) abscess or other evidence of organ/space infection detected by gross anatomical examination, histopathological examination, or imaging; and (4) diagnosis by a surgeon or attending physician. In this study, the final diagnosis of organ/space SSI was determined by comprehensive review of clinical signs postoperative fever, abdominal signs, inflammatory markers, imaging findings, interventional or intraoperative findings, in addition to microbiological results when available. Results from tNGS or CMTs of drainage fluid were considered adjunctive microbiological evidence and were not used as the sole criterion for diagnosing organ/space SSI.

Definitions of clinically relevant postoperative pancreatic fistula (CR-POPF), delayed gastric emptying (DGE), and post-pancreatectomy hemorrhage (PPH) were adopted from the standard classifications proposed by the International Study Group of Pancreatic Surgery (ISGPS) (Wente et al., 2007a,b; Bassi et al., 2017). In addition, the diagnostic criteria for bile leakage (BL) were applied in line with the recommendations of the International Study Group of Liver Surgery (Koch et al., 2011).

### Statistical analysis

All statistical analyses were performed using SPSS (version 26.0) for Windows (SPSS Inc.). Categorical variables were described as frequency and percentage, and compared using the Pearson χ^2^ test or Fisher’s exact test as appropriate. Continuous variables with normal distribution were presented as the mean ± standard deviation (SD) and compared using the independent-samples *t*-test. Non-normally distributed continuous variables were expressed as the median (interquartile range, IQR) and analyzed by the Mann–Whitney *U* test. To explore factors associated with tNGS positivity, variables with a *p* value <0.10 in univariate analyses were entered into a multivariate logistic regression model. Odds ratios (OR) with corresponding 95% confidence intervals (CI) were calculated. The paired McNemar χ^2^ test was utilized to compare the diagnostic efficiency of tNGS and CMTs. The overall pathogen detection rate was defined as the proportion of patients with a positive microbiological result among all enrolled patients. Sensitivity, specificity, positive predictive value, and negative predictive value were calculated using the final clinical diagnosis of organ/space SSI as the reference standard. Receiver operating characteristic (ROC) curve analysis was performed, and the area under the ROC curve (AUC) was calculated to assess the diagnostic accuracy of tNGS and CMTs. A two-sided *p* < 0.05 was considered statistically significant.

## Results

### Patient characteristics

A total of 73 patients who underwent biliary-pancreatic surgery were enrolled in this study. The cohort consisted of 33 (45.2%) males and 40 (54.8%) females, with a mean age of 60.6 years. Among the 73 patients, 23 (31.5%) were diagnosed with preoperative jaundice, and 15 (20.5%) had undergone preoperative biliary drainage (PBD). Pancreatic surgery was performed in 59 (80.8%) patients, including pancreaticoduodenectomy (*n* = 42), distal pancreatectomy (*n* = 15) and total pancreatectomy with splenectomy (*n* = 2), whereas biliary surgery was performed in 14 patients (19.2%), including hepatectomy with biliary reconstruction (*n* = 6), extrahepatic bile duct resection with choledochojejunostomy (*n* = 3), and cholecystectomy with common bile duct exploration (*n* = 5). The incidence of postoperative organ/space SSI was 36.9% (27/73), followed by a 21.9% (16/73) rate of CR-POPF and 9.6% (7/73) of DGE. The positivity rates for tNGS and CMTs were 52.1 and 39.7%, respectively (Table 1).

**Table 1:** Patients characteristics.

Characteristics	All patients (*n* = 73)
Age (mean ± SD), years	60.6 ± 14.5
BMI (mean ± SD), kg/m^2^	21.9 ± 2.9
Gender, *n* (%)	
Male	33 (45.2)
Female	40 (54.8)
Hypertension, *n* (%)	23 (31.5)
DM, *n* (%)	12 (16.4)
Smoking, *n* (%)	17 (23.3)
Drinking, *n* (%)	13 (17.8)
Preoperative jaundice, *n* (%)	23 (31.5)
PBD, *n* (%)	15 (20.5)
Surgery procedure, *n* (%)	
Pancreatic surgery	59 (80.8)
Pancreaticoduodenectomy	42 (57.5)
Distal pancreatectomy	15 (20.5)
Total pancreatectomy with splenectomy	2 (2.7)
Biliary surgery	14 (19.2)
Hepatectomy with biliary reconstruction	6 (8.2)
Extrahepatic bile duct resection	3 (4.1)
Cholecystectomy with common bile duct exploration	5 (6.8)
Operation time (median, IQR), min	255.0 (175.0–357.5)
Blood loss volume (median, IQR), ml	300.0 (200.0–600.0)
WBC (median, IQR), ×10^9^/L	12.1 (9.8–15.9)
PLT (median, IQR), ×10^9^/L	192.0 (132.0–243.5)
Alb (mean ± SD), g/L	32.9 ± 5.2
CRP (median, IQR), mg/L	44.0 (31.1–68.4)
Hemoglobin (mean ± SD), g/L	108.1 ± 17.8
Organ/space SSI, *n* (%)	27 (36.9)
CR-POPF, *n* (%)	16 (21.9)
DGE, *n* (%)	7 (9.6)
BL, *n* (%)	3 (4.1)
PPH, *n* (%)	3 (4.1)
tNGS positive, *n* (%)	38 (52.1)
CMTs positive, *n* (%)	29 (39.7)

IQR, interquartile range; SD, standard deviation; BMI, body mass index; DM, diabetes mellitus; PBD, preoperative biliary drainage; Alb, albumin; CRP, C-reactive protein; WBC, white blood cell; PLT, platelet; SSI, surgical site infection; CR-POPF, clinically relevant postoperative pancreatic fistula, BL, biliary leakage; PPH, post-pancreatectomy hemorrhage; tNGS, targeted next generation sequencing; CMTs, conventional microbiological tests.

### Factors associated with tNGS positivity

All patients were categorized into two groups as tNGS negative group (*n* = 35, 47.9%) and tNGS positive group (*n* = 38, 52.1%). The univariate analysis revealed significant differences between the two groups in terms of age, diabetes mellitus (DM), preoperative jaundice, and PBD. In the exploratory multivariate analysis, advanced age and PBD were associated with tNGS positivity (Table 2).

**Table 2:** Univariate and multivariate analysis of positive tNGS.

Variables	tNGS negative (*n* = 35)	tNGS positive (*n* = 38)	*P*	OR (95%CI)	*P*
Age (mean ± SD), years	56.1 ± 15.9	64.84 ± 11.8	0.009	1.052 (1.005–1.100)	0.029
BMI (mean ± SD), kg/m^2^	21.6 ± 3.2	22.3 ± 2.6	0.327		
Gender, *n* (%)			0.699		
Male	15 (42.9)	18 (47.4)			
Female	20 (57.1)	20 (52.6)			
Hypertension, *n* (%)	8 (22.9)	15 (39.5)	0.127		
DM, *n* (%)	2 (5.7)	10 (26.3)	0.018	5.092 (0.799–32.432)	0.085
Smoking, *n* (%)	6 (17.1)	11 (28.9)	0.233		
Drinking, *n* (%)	4 (11.4)	9 (23.7)	0.172		
Preoperative jaundice, *n* (%)	6 (17.1)	17 (44.7)	0.011	1.899 (0.327–11.026)	0.475
PBD, *n* (%)	2 (5.7)	13 (34.2)	0.003	9.411 (1.060–83.524)	0.044
WBC (median, IQR), ×10^9^/L	11.2 (9.5–14.7)	12.1 (10.2–16.2)	0.483		
PLT (median, IQR), ×10^9^/L	196.0 (140.0–233.0)	188.5 (124.0–248.5)	0.707		
Alb (mean ± SD), g/L	33.2 ± 4.2	32.6 ± 6.1	0.633		
CRP (median, IQR), mg/L	39.7 (29.9–60.8)	51.1 (31.9–83.6)	0.237		
Hb (mean ± SD), g/L	108.9 ± 16.1	107.3 ± 19.3	0.687		
Surgery procedure, *n* (%)			0.864		
Pancreatic surgery	28 (80.0)	31 (81.6)			
Biliary surgery	7 (20.0)	7 (18.4)			
Operation time (median, IQR), min	220.0 (155.0–355.0)	275.0 (182.5–363.8)	0.180		
Blood loss volume (median, IQR), ml	200.0 (100.0–400.0)	400.0 (200.0–800.0)	0.012	1.001 (1.00–1.002)	0.063

tNGS, targeted next generation sequencing; IQR, interquartile range; SD, standard deviation; OR, odds ratio; CI, confidence interval; BMI, body mass index; DM, diabetes mellitus; PBD, preoperative biliary drainage; Alb, albumin; CRP, C-reactive protein; WBC, white blood cell; PLT, platelet.

### Comparison of diagnostic performance between tNGS and CMTs

Among the 73 patients, 26 (35.6%) yielded positive results for both tNGS and CMTs, 32 (43.8%) were negative by both methods. 12 (16.4%) patients were positive by tNGS but negative by CMTs, and 3 (4.1%) patients were positive by CMTs but negative by tNGS. The microorganisms identified by CMTs in the 3 CMTs-positive/tNGS-negative cases were *Staphylococcus epidermidis* in two patients and *Staphylococcus haemolyticus* in one patient. Among the 32 patients negative by both tNGS and CMTs, 2 were finally diagnosed with organ/space SSI. The overall microbiological positivity rate was higher for tNGS than CMTs (Table 3). As for organ/space SSI, tNGS demonstrated higher sensitivity (92.6% vs. 66.7%), positive predictive value (65.8% vs. 62.1%), and negative predictive value (94.3% vs. 79.5%) than CMTs, but lower specificity (71.7% vs. 76.1%) (Table 4).

**Table 3:** Comparison of microbiological positivity between tNGS and CMTs.

tNGS (cases)	CMTs (cases)		Total (cases)
	Negative	Positive	
Negative	32	3	35
Positive	12	26	38
Total (Cases)	44	29	73
*P*	0.035		

tNGS, targeted next generation sequencing; CMTs, conventional microbiological tests.

**Table 4:** Diagnostic performance of tNGS and CMTs for organ/space SSI.

Detection method	Sensitivity (%)	Specificity (%)	Positive predictive value (%)	Negative predictive value (%)
CMTs	66.7	76.1	62.1	79.5
tNGS	92.6	71.7	65.8	94.3

tNGS, targeted next generation sequencing; CMTs, conventional microbiological tests; SSI, surgical site infection.

**Figure 1:**
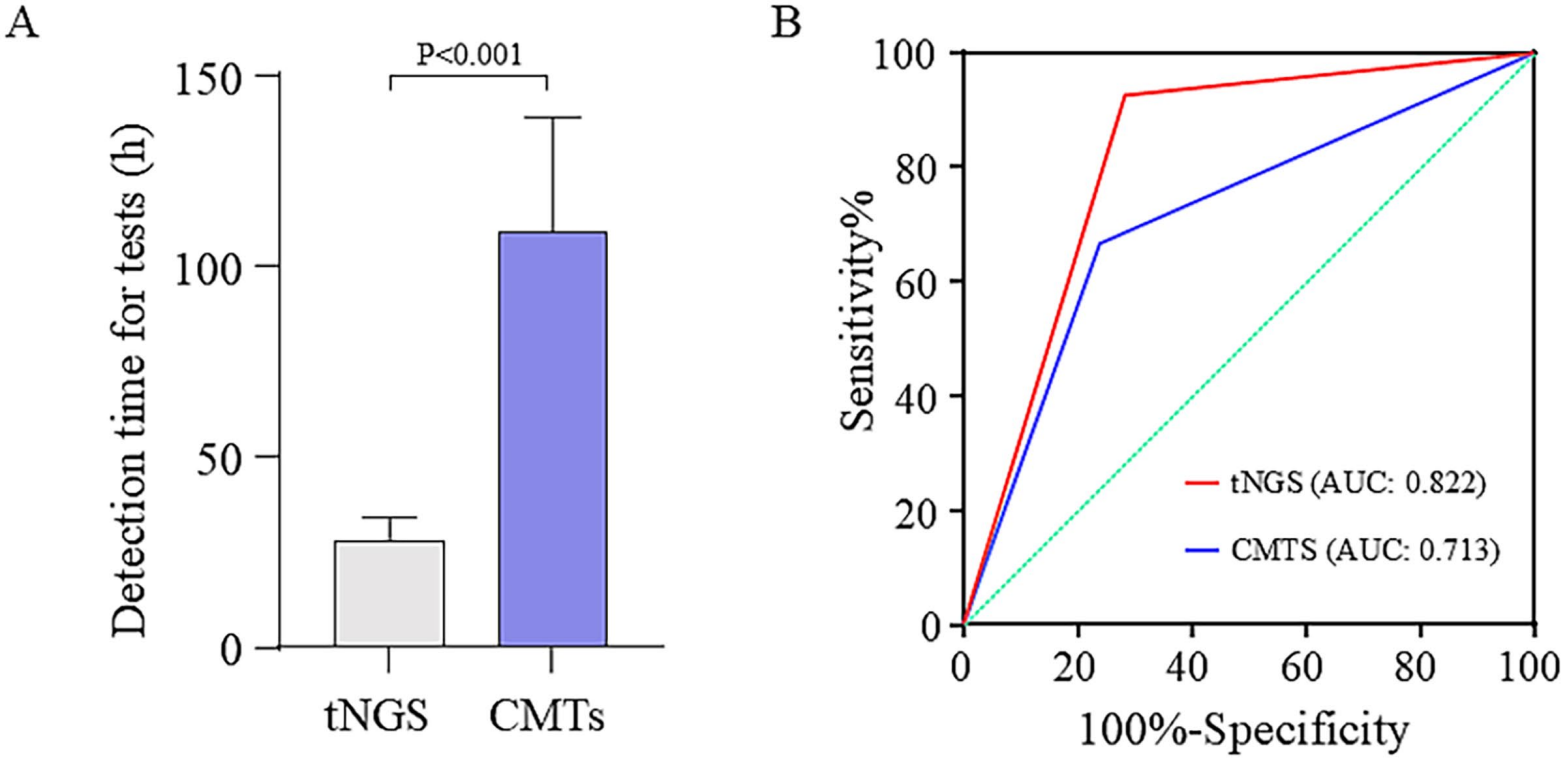
Comparison of clinical diagnostic performance between tNGS and CMTs. **(A)** The detection time of tNGS was significantly less than that of CMTs; **(B)** ROC curves for different diagnostic methods. tNGS, targeted next generation sequencing; CMTs, conventional microbiological tests; AUC, area under the curve.

Moreover, tNGS exhibited an average pathogen detection time of 28.1 ± 6.1 h, whereas bacterial culture had an average time of 109.1 ± 30.0 h. The significantly lower average time of tNGS than that of bacterial culture (*p* < 0.001) underscores its timeliness in pathogen detection (Figure 1). Diagnostic performance of both methods was further assessed using ROC analysis, which revealed an AUC of 0.822 (95% CI: 0.722–0.921) for tNGS, compared to 0.713 (95% CI: 0.587–0.840) for CMTs (Figure 1).

### Pathogen identification by tNGS and CMTs

**Figure 2:**
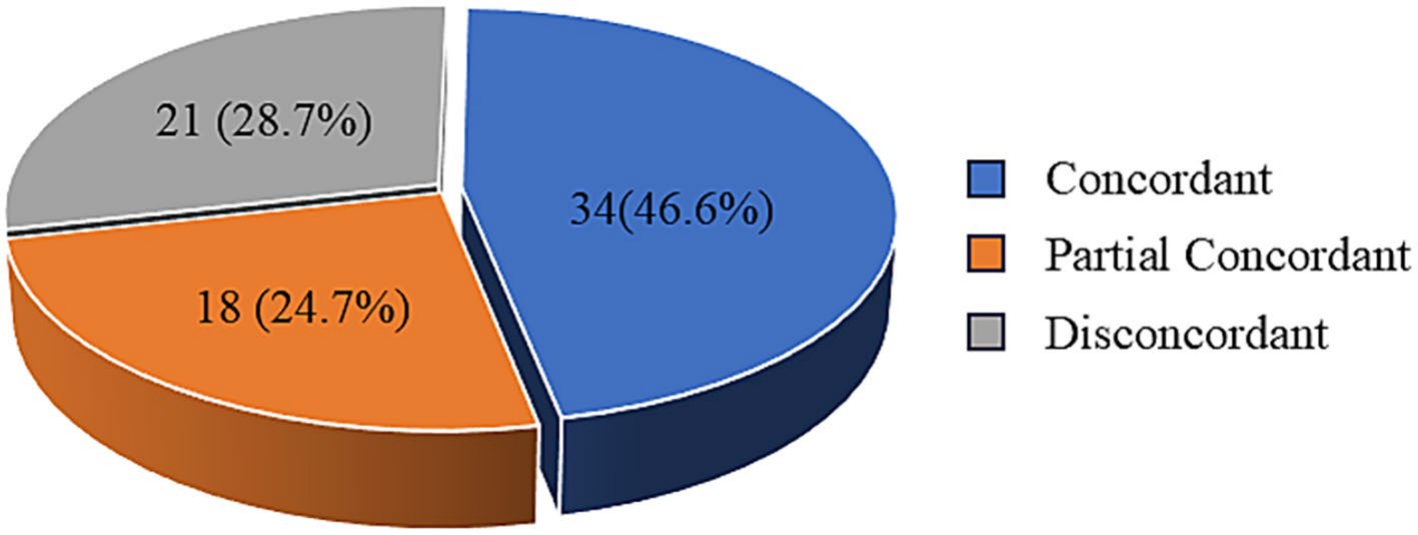
Concordance between tNGS and CMTs. tNGS, targeted next generation sequencing; CMTs, conventional microbiological tests.

As shown in Figure 2, 34 (46.6%) showed complete concordant, 21 (28.7%) showed complete disconcordant, and 18 (24.7%) showed partial concordant between CMTs and tNGS. The microbial spectra detected by tNGS and CMTs were displayed in Table 5. CMTs identified 12 pathogenic species, including 11 bacterial species and 1 fungal species. The pathogen spectrum revealed that *Klebsiella pneumoniae* (*n* = 9, 12.3%), *Staphylococcus epidermidis* (*n* = 8, 10.9%), *Staphylococcus haemolyticus* (*n* = 6, 8.2%), *E. coli* (*n* = 4, 5.5%) and *Candida albicans* (*n* = 3, 4.1%) were the leading bacterial pathogens followed by *Enterococcus faecium* (*n* = 2, 2.7%), *Enterococcus faecalis* (*n* = 2, 2.7%), *Enterobacter cloacae* (*n* = 1, 1.4%), *Acinetobacter baumannii* (*n* = 1, 1.4%), *Aeromonas hydrophila* (*n* = 1, 1.4%), *Staphylococcus aureus* (*n* = 1, 1.4%), and *Streptococcus anginosus* (*n* = 1, 1.4%).

**Table 5:** Microbial spectrum detected by tNGS and CMTs.

Microorganisms	CMTs (*n* = 73)	tNGS (*n* = 73)
*Klebsiella pneumoniae*, *n* (%)	9 (12.3)	17 (23.3)
*E. coli*, *n* (%)	4 (5.5)	9 (12.3)
*Enterobacter cloacae*, *n* (%)	1 (1.4)	2 (2.7)
*P. aeruginosa*, *n* (%)	0 (0.0)	1 (1.4)
*Acinetobacter baumannii*, *n* (%)	1 (1.4)	2 (2.7)
*Veillonella parvula*, *n* (%)	0 (0.0)	3 (4.1)
*Haemophilus influenzae*, *n* (%)	0 (0.0)	3 (4.1)
*Citrobacter freundii*, *n* (%)	0 (0.0)	1 (1.4)
*Aeromonas dhakaensis*, *n* (%)	0 (0.0)	1 (1.4)
*Aeromonas hydrophila*, *n* (%)	1 (1.4)	0 (0.0)
*Prevotella intermedia*, *n* (%)	0 (0.0)	1 (1.4)
*Enterococcus faecium*, *n* (%)	2 (2.7)	12 (16.4)
*Enterococcus faecalis*, *n* (%)	2 (2.7)	7 (9.6)
*Staphylococcus epidermidis*, *n* (%)	8 (10.9)	6 (8.2)
*Staphylococcus haemolyticus*, *n* (%)	6 (8.2)	0 (0.0)
*Staphylococcus aureus*, *n* (%)	1 (1.4)	3 (4.1)
*Streptococcus anginosus*, *n* (%)	1 (1.4)	2 (2.7)
*Streptococcus pneumoniae*, *n* (%)	0 (0.0)	1 (1.4)
*Actinomyces viscosus*, *n* (%)	0 (0.0)	2 (2.7)
*Finegoldia magna*, *n* (%)	0 (0.0)	1 (1.4)
*Candida albicans*, *n* (%)	3 (4.1)	3 (4.1)
*Candida glabrata*, *n* (%)	0 (0.0)	1 (1.4)
*Candida tropicalis*, *n* (%)	0 (0.0)	3 (4.1)
Parvovirus, *n* (%)	0 (0.0)	2 (2.7)
Herpesvirus, *n* (%)	0 (0.0)	7 (9.6)

tNGS, targeted next generation sequencing; CMTs, conventional microbiological tests.

In contrast, tNGS detected a broader microbial spectrum, including 18 bacterial species, 3 fungal species, and 2 viral targets. The most frequently detected bacterial organisms by tNGS were *Klebsiella pneumoniae* (*n* = 17, 23.3%), *Enterococcus faecium* (12, 16.4%), *E. coli* (*n* = 9, 12.3%), *Enterococcus faecalis* (*n* = 7, 9.6%) and *Staphylococcus epidermidis* (*n* = 6, 8.2%). The detected fungal organisms included *Candida albicans* (*n* = 3, 4.1%), *Candida tropicalis* (*n* = 3, 4.1%) and Candida glabrata (*n* = 1, 1.4%). Viral sequences were detected by tNGS in 7 (9.6%) patients, all of whom were positive for herpesvirus; among these patients, 2 (2.7%) were also positive for parvovirus. These viral detections were reported as microbiological findings rather than definitive causative pathogens of organ/space SSI.

## Discussion

In the current study, we evaluated the clinical performance of tNGS for pathogen detection in postoperative abdominal drainage fluid after biliary-pancreatic surgery. Our findings showed that early postoperative tNGS provided a higher detection rate and substantially shorter detection time than routine CMT-based surveillance, supporting its role as a useful adjunctive microbiological tool for early pathogen assessment associated with organ/space SSI after biliary-pancreatic surgery.

The favorable diagnostic performance of tNGS in our cohort was mainly reflected by its higher sensitivity (92.6% vs. 66.7%) and overall pathogen detection rate (52.1% vs. 39.7%) compared to CMTs, which is consistent with previous studies evaluating molecular diagnostic methods in infectious diseases (Flurin et al., 2021; Gaston et al., 2022). Consistent with the known microbiological characteristics of organ/space SSI after pancreatobiliary surgery (Yang et al., 2021; Ellis et al., 2023; Zhu et al., 2023; Ciprani et al., 2024), both methods frequently detected organisms such as *Klebsiella pneumoniae*, *E. coli*, Enterococcus species, and Staphylococcus species. Notably, tNGS revealed a broader microbial spectrum (23 distinct species compared to 12 by CMTs). This enhanced performance may be explained by the culture-independent nature of tNGS, which enables the detection of slow-growing, fastidious, low-abundance, or difficult-to-culture organisms in the complex polymicrobial environment of the postoperative abdominal cavity. Following biliary-pancreatic surgery, especially procedures involving biliary-pancreatic reconstruction, the operative field may be exposed to endogenous gastrointestinal and biliary flora. This exposure may be more pronounced in patients with PBD, in whom retrograde biliary colonization is common (Ellis et al., 2023; Zhu et al., 2023). In such settings, CMTs may fail to recover the full microbial spectrum due to polymicrobial competition, overgrowth by the rapidly proliferating bacteria (e.g., *Klebsiella pneumoniae* and *E. coli*) during *in vitro* cultivation (Sartelli et al., 2017), and the influence of perioperative antimicrobial exposure. By directly amplifying microbial nucleic acids, tNGS can partly overcome these culture-dependent limitations, which may explain the higher detection rates of *Enterococcus faecium*, *Enterococcus faecalis*, and Candida species in our cohort. Recognizing these clinically relevant organisms is important, as these organisms may contribute to postoperative intra-abdominal abscess formation and secondary complications (Shogan et al., 2015; Belmouhand et al., 2018; Chang et al., 2026).

Nevertheless, drainage-fluid microbiology should be interpreted with caution. Although abdominal drains provide clinically accessible samples for microbiological assessment, they do not represent a completely sterile sampling environment, especially after prolonged postoperative indwelling. Therefore, microorganisms detected by either tNGS or CMTs should not be automatically regarded as causative pathogens of organ/space SSI. This consideration is particularly important for skin commensals, such as coagulase-negative Staphylococci, including *Staphylococcus epidermidis*, which may reflect external contamination or drain colonization rather than true intra-abdominal infection in some cases. Similarly, viral nucleic acid fragments detected by tNGS may represent latent viral reactivation, subclinical shedding, or molecular background rather than clinically relevant organ/space SSI (Simner et al., 2018). No antiviral therapy was initiated solely on the basis of viral reads detected by tNGS in our cohort. Accordingly, microbiological findings in this study were interpreted as adjunctive etiological evidence and required clinical adjudication. Although negative results by both tNGS and CMTs may suggest a low microbial burden in drainage fluid, they do not completely exclude organ/space SSI and should be interpreted together with clinical, laboratory, imaging, and drainage-related findings, particularly when infection remains clinically suspected.

The shorter turnaround time represents a potential clinical advantage of tNGS. Organ/space SSI following biliary-pancreatic surgery may progress rapidly and can be associated with CR-POPF, PPH and severe sepsis (Shogan et al., 2015; Yamashita et al., 2015; Demir et al., 2020). CMTs often require several days to provide pathogen identification and antimicrobial susceptibility results, during which clinicians frequently rely on empirical antimicrobial therapy. In contrast, early tNGS can provide microbiological information within approximately 28 h, which may help clinicians reassess empirical treatment strategies earlier. This is particularly relevant in patients with persistent fever, elevated inflammatory markers, clinical deterioration, abnormal drainage characteristics, or imaging evidence of intra-abdominal fluid collection. However, tNGS should not be used in isolation to escalate or de-escalate antimicrobial therapy. Treatment decisions should rely on the overall clinical status and, when available, antimicrobial susceptibility results from conventional culture.

The relatively high negative predictive value observed for tNGS may provide additional clinical information in the postoperative assessment of suspected infection. A negative tNGS result may lower the likelihood of a high microbial burden in drainage fluid and may provide supportive information when clinicians reassess the probability of organ/space SSI. However, this should not be interpreted as sufficient evidence to exclude infection in all situations. Similarly, antimicrobial de-escalation or drain removal should not be determined by tNGS results alone. In HPB surgery, drain management depends on multiple factors, including drainage volume and appearance, amylase or bilirubin levels in the drainage fluid, the presence or absence of pancreatic fistula or bile leakage, imaging findings, and the patient’s overall postoperative course. Thus, tNGS is best regarded as an additional microbiological layer that complements, rather than replaces, established surgical and clinical assessment.

Furthermore, in the exploratory multivariate analysis, advanced age and PBD were associated with tNGS positivity. This finding is consistent with previous studies. PBD may compromise the mechanical barrier of the sphincter of Oddi, facilitate the retrograde colonization of the biliary tract by enteric flora, and increase the likelihood of operative-field contamination (Yang et al., 2025). Similarly, advanced age is frequently associated with diminished local immunity and altered mucosal barriers, further increasing the likelihood of postoperative bacterial colonization (Kaye et al., 2005; Thevaranjan et al., 2018). These exploratory findings suggest that patients with advanced age or PBD may represent a subgroup in whom early tNGS testing could be considered, although validation in larger prospective cohorts is needed.

This study has several limitations. First, the retrospective, single-center design with a relatively small sample size may limit the generalizability of the findings. Second, intraoperative bile cultures were not uniformly available and analyzed in this retrospective cohort. Future prospective studies incorporating bile cultures and sterile intra-abdominal specimens may help clarify the distinction between true organ/space SSI, biliary colonization, drain colonization, and contamination. Third, although tNGS enables rapid detection, it does not provide phenotypic antimicrobial susceptibility results, which remain a vital advantage of CMTs. Finally, the higher cost of tNGS may limit its routine use and may be better justified in selected patients with a high suspicion of organ/space SSI or when CMTs is inconclusive.

In conclusion, early postoperative tNGS may serve as a useful adjunctive microbiological tool for pathogen detection of organ/space SSI following biliary-pancreatic surgery. Compared with CMTs, tNGS showed a higher pathogen detection rate, higher sensitivity and negative predictive value, and shorter turnaround time. However, tNGS results should be interpreted in conjunction with clinical manifestations, rather than being used as a standalone diagnostic criterion for organ/space SSI. These findings support the selective use of tNGS as an early etiological assessment tool to assist postoperative organ/space SSI management and antimicrobial decision-making.

## Data Availability

The original contributions presented in the study are included in the article/supplementary material, further inquiries can be directed to the corresponding authors.
